# Endoscopic features of a serrated tubulovillous adenoma in the sigmoid colon

**DOI:** 10.1055/a-2652-3406

**Published:** 2025-08-08

**Authors:** Stefan Mitev, Jean Hanna Bakraji, Diana Kyoseva

**Affiliations:** 1 266309 Gastroenterology Clinic, University Hospital St Ivan Rilski, Sofia, Bulgaria; 2 Department of General and Clinical Pathology, University Hospital Alexandrovska, Medical University Sofia, Sofia, Bulgaria

Serrated tubulovillous adenoma (STVA) is a rare type of colorectal polyp that exhibits characteristics of both serrated lesions and conventional adenomas. These polyps show frequent KRAS mutations and CpG island methylation. Therefore, they represent potential precursors to KRAS-mutated, microsatellite-stable colorectal carcinomas. We present a case clearly showing a demarcation line between the two distinct components of an STVA.

A 69-year-old man, with a past medical history notable only for a remote appendectomy, presented for a screening colonoscopy ( Video 1 ). Over 20 flat lesions (3–20 mm) with optical features of hyperplastic and serrated polyps were found predominantly in the proximal colon. These findings were consistent with serrated polyposis syndrome. Additionally, a subpedunculated polyp, approximately 15 mm in size, was found in the sigmoid colon ( Fig. 1 ). Close examination with TXI ( Fig. 2 ) and NBI ( Fig. 3 ) revealed a distinct demarcation line separating two visually different areas within the polyp. The presence of a demarcation line was initially interpreted as a possible superficial submucosal invasion. The polyp was completely removed by hot snare polypectomy after placement of a detachable snare around its base ( Fig. 4 ).

Endoscopic features of a serrated tubulovillous adenoma in the sigmoid colon.Video 1

**Fig. 1:**
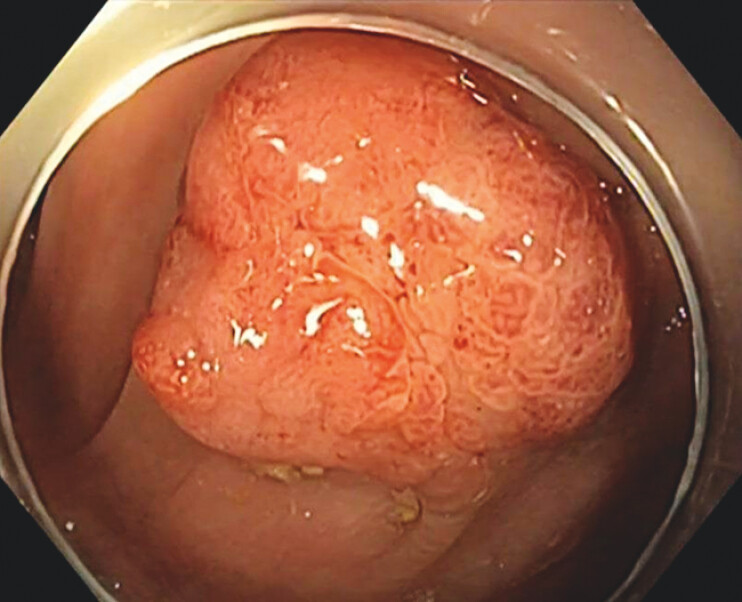
Texture and color enhancement imaging (TXI) view of a subpedunculated polyp in the sigmoid colon.

**Fig. 2:**
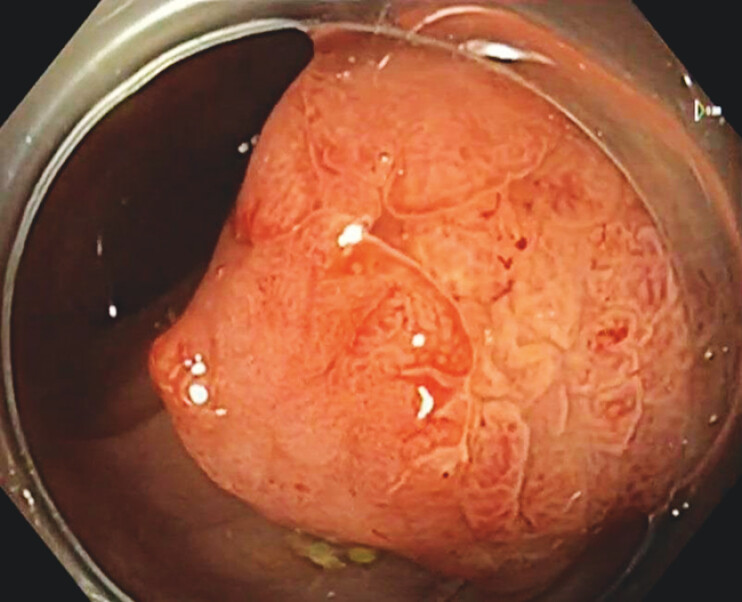
Closer inspection with TXI shows a clear demarcation line between two distinct areas of the polyp.

**Fig. 3:**
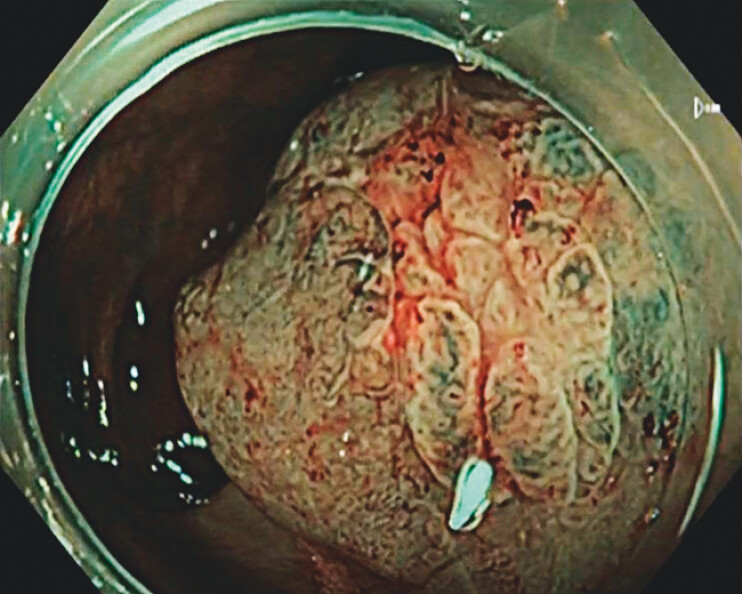
Narrow band imaging (NBI) of the demarcation line.

**Fig. 4:**
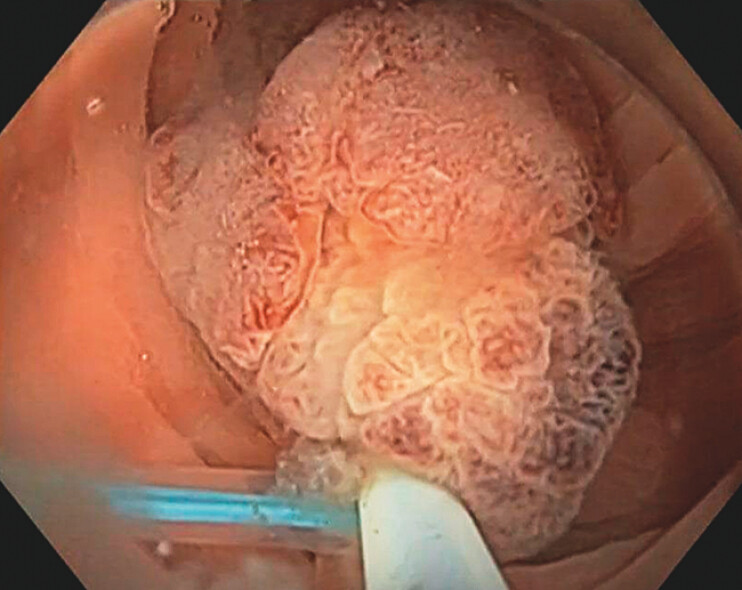
Hot snare polypectomy of the serrated tubulovillous adenoma.

Gross pathological examination of the resected specimen revealed a lobulated polypoid lesion with two distinctly colored regions: one whitish and the other light brown. Histopathology confirmed two clearly delineated regions: one with serrated features and the other corresponding to a tubulovillous adenoma with low-grade dysplasia. The specimen contained >25% villous component, >50% morphological serration, and <10% slit-like serrations ( Fig. 5 ), fulfilling the diagnostic criteria for an STVA 1 .

**Fig. 5:**
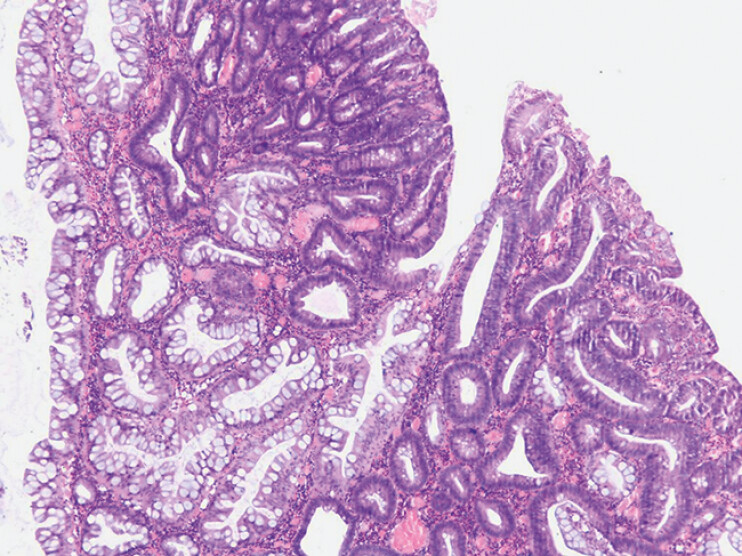
Two distinct regions within the polyp: one with serrated features (predominantly on the left side of the image) and the other corresponding to a tubulovillous adenoma with low-grade dysplasia (predominantly on the right side of the image).

This case, particularly within the context of serrated polyposis syndrome, highlights the importance of optical diagnosis for colorectal polyp characterization. Recognizing the demarcation line between the serrated and tubulovillous components of an STVA may aid in initial diagnosis prior to histopathological confirmation.

Endoscopy_UCTN_Code_CCL_1AD_2AB

## Notes

Endoscopy E-Videos https://eref.thieme.de/e-videos
*E-Videos* is an open access online section of the journal *Endoscopy* , reporting on interesting cases and new techniques in gastroenterological endoscopy. All papers include a high-quality video and are published with a Creative Commons CC-BY license. Endoscopy E-Videos qualify for HINARI discounts and waivers and eligibility is automatically checked during the submission process. We grant 100% waivers to articles whose corresponding authors are based in Group A countries and 50% waivers to those who are based in Group B countries as classified by Research4Life (see: https://www.research4life.org/access/eligibility/ ). This section has its own submission website at https://mc.manuscriptcentral.com/e-videos .
